# Health Risks Associated with Oil Pollution in the Niger Delta, Nigeria

**DOI:** 10.3390/ijerph13030346

**Published:** 2016-03-21

**Authors:** Jerome Nriagu, Emilia A. Udofia, Ibanga Ekong, Godwin Ebuk

**Affiliations:** 1 Department of Environmental Health Sciences, School of Public Health, University of Michigan, Ann Arbor, MI 48109, USA; 2 Department of Community Health, School of Public Health, University of Ghana, P. O. Box LG 13, Legon, Ghana; emiliaudf@yahoo.com or eaudofia@ug.edu.gh; 3 Department of Community Health, Faculty of Clinical Sciences, University of Uyo, P.M.B. 1017, Uyo, Akwa Ibom, Nigeria; ibangeky@yahoo.com; 4 Department of Public Health Services, Akwa Ibom Ministry of Health Headquarters, P.M.B. 1030, Uyo, Akwa Ibom, Nigeria; drjameg@gmail.com

**Keywords:** environmental worry, environmental hazard annoyance, risk perception, risk tolerance, functional capacity limitation, health symptoms index, rumination, chronic stress

## Abstract

*Background*: Although there is considerable public concern about the environmental impacts of oil pollution in the Niger Delta of Nigeria, actual evidence on the pathological and psychological effects in the health of local communities is minimally known. We sought to associate the perspective measures of exposure to oil pollution with health outcomes (inventory of health symptoms and functional capacity limitations) and determine how emotional reactions to environmental risks moderate these health outcomes. *Method*: The study was conducted with 600 participants selected from five local government areas in Akwa Ibom State where oil pollution is rampant. A structured questionnaire was used to collect the data on the respondents’ exposure to oil pollution, self-rated health and disease symptoms, perception of risk of exposure and emotional reactions to local oil pollution. *Results*: Most of the participants lived in areas with visible oil pollution and/or near gas flaring facilities and regularly suffered direct exposure to oil in their environment. High level of emotional distress was a part of everyone's life for the study population. Risk perception in the study area was mediated, to a large extent, by dreaded hazards (catastrophic fears of pipeline explosions and oil spill fire), visual cues (gas flares and smoke stacks) and chemosensory cues (off-flavor in drinking water). The exposure metrics were found to be significant predictors of the health effects and influencing factors (emotional reactions). Multi-levels models suggest that at the individual level, the demographic variables and direct contact with oil pollution were important mediators of functional capacity limitation. At the community level, emotional distress from fear of the sources of exposure was an important mediator of the health symptoms. *Conclusions*: This study documents high levels of disease symptoms and environmental distress (worry, annoyance and intolerance) associated with oil pollution in the Niger Delta areas of Nigeria. It highlights the need for some intervention to ameliorate the psychological distress associated with living under such environmental adversity.

## 1. Introduction

Nigeria is the largest oil producer in Africa and the eleventh largest producer of crude oil in the world. Prospecting and extraction of petroleum occur in over 50% of the Niger Delta region, resulting in a cornucopia of access roads, pipelines, wells, gas flaring, dredged spoils and flow stations that are often located near homes, schools, farms, and within communities [1]. Oil spills are common throughout the area, as a consequence of pipeline corrosion, poor maintenance of infrastructure, spills or leaks at the well heads, human error, theft of oil and intentional vandalism [2,3]. The amount of oil that is spilled in the Niger delta is unknown. A report by Jernelov [4] estimated that the total spillage was between 9 and 13 million barrels over 50 years—roughly 1.5 million tons per year, equivalent to one Exxon-Valdez spill annually for half a century. The collective impacts of these pervasive massive spills on the environment and local inhabitants are worsened by seasonal floods which transfer the oil pollution to farmlands and occupied areas [5]. Currently, hundreds of thousands of people who live in the Niger delta are being exposed to oil contamination near their homes, farm lands, fishing grounds and in their drinking water and foods but the consequences of such exposure on their health are unknown [1].

Most of the human studies in the Niger Delta have been focused on the caustic relationship between the oil pollution and poverty in the region and the social tension between the people and the oil companies [6,7,8]. Although health problems are cited in these studies to support the contention that concern for a clean environment is a reason for community-industry conflicts, there have been few systematic health studies to back up such claims [1,5]. Reliable information on the effects of oil pollution on the physiological health of people in the Niger Delta is very limited (reviewed by Nriagu [1]; Ordinioha and Brisibe [6]), and even less is known about the psychological effects of living with such environmental adversity [7,9]. The first goal of this study is to determine the prevalence and correlates of two health outcome measures (namely, functional capacity limitation and health symptoms) and emotional distress (environmental worry, environmental annoyance and environmental intolerance) in an area of the Niger Delta where oil pollution is rampant. The second goal is to explore the perception of environmental risks by the local population and its influence on the emotional distress. The third goal is focused on how the relationships between exposure to oil pollution and the health outcome measures are mediated by: (i) risk perception; and (ii) emotional distress in the study population. The interrelations between oil pollution, perceived risks of the pollution, emotional distress, physical activity limitations and health symptoms have not been studied previously in the oil polluted communities of Nigeria or other countries. This study with a large sample size has the statistical power to provide meaningful inferences from the results. A better understanding of the influencing factors on health outcome measures from exposure to oil is important in developing intervention strategies to minimize the psychopathological ill health in oil-producing areas of the country.

The effects of *large accidental* oil spills on the environment and human health have been extensively studied in different parts of the world (reviewed by Aguilera *et al.* [10]). These industrial disasters are invariably marked by identifiable endpoints followed by the recovery phase and the period after an oil spill is usually followed by concerted effort by external agencies, volunteers and residents working together to clean up the environment, rebuild the community and support individuals [11,12,13,14]. By contrast, oil pollution in the Niger Delta is an ongoing chronic disaster and an environmental adversity with no end in sight, and entails little or no support for communities and individuals that are affected [8,15]. The chronic nature of the oil pollution and its associated environmental and social impacts may have an insidious impact on one’s physical health (sustained systemic toxicity by oil-related contaminants) and mental health (such as increased risk for high levels of distress) which are different from those of discrete traumatic events [16]. A goal of this study is to develop new instruments for assessing the multidimensional elements of exposure and health outcomes in communities with oil pollution. The comorbidity of physiological and psychological health effects due to chronic exposure to sustained environmental pollution by oil has not received much scientific attention and this study is aimed at helping to fill this gap in knowledge. We are not aware of any previous study.

## 2. Methodology

### 2.1. Study Area

Akwa Ibom State (AKS), the site for this study, is located in the Niger Delta region of Nigeria. It has a population of 3.9 million people spread across 31 local government areas (LGAs) which are mainly rural. It is currently the largest oil producing state in Nigeria and is also believed to have one of the largest natural gas deposits in the country [17]. Oil exploration and production have gone on for decades in the state which has been scarred by numerous oil spills, pipeline explosions and oily waste discharges.

### 2.2. Study Population

The cross sectional study was conducted from December 2013 to January 2014. The target population consisted of adults in coastal communities in five local government areas (LGAs) of AKS (namely Eket, Onna, Ibeno, Mbo and Oron). Healthy individuals aged 18 years or older who had lived in any of the LGAs for no less than five years were recruited from designated health centers in each LGA. The interviewers targeted the medical staff of the clinics, parents who had brought their children to the clinics for medical care and individuals who had accompanied their relatives and/or friends to the clinics. Since the oil companies provide free medical care to their employees, there was no incentive for employees of an oil company to seek medical services at the clinics used in this study. We therefore believe that few of the participants were employees of the oil company. Individuals who were sick, mentally challenged, did not meet the residency requirement or were unwilling to participate were excluded from the study. Only one member of each household was eligible to participate.

Local interviewers were selected and trained for two days on the purpose of the study, field protocol, questionnaire administration, translation and back-translation of the instrument, and ethical issues. The instrument was pre-tested for language clarity during the training and modified based on the feedback. A one-day briefing was also provided to the staff of the health centers from each LGA designated by the AKS Ministry of Health to assist with recruitment of participants in the study.

At each health center, the interviewers worked with the designated local staff to identify potential participants. Eligible individuals willing to participate were interviewed in a secluded space provided at each health center to ensure they were comfortable and less likely to be influenced by other people around them. The questionnaire took an average of 30–40 min to administer. Because of fear and civil unrest in the study area and to ensure honest answers from participants, the survey was anonymized—we did not ask for participants’ names, phone numbers, addresses or any identifying information.

After an interview, height measurements in meters (m) were obtained using a meter stick. Individuals were made to remove their shoes and any head gear that would bias the measurements. They stood looking forward with their heels against the wall, shoulders squared and arms by their sides. Weight measurements were taken in kilograms (kg) using portable bathroom scales with a precision of 0.5 kg. Individuals were required to remove their shoes and stand on the scale for measurements to be taken. The scales were standardized after each measurement. Both measurements were used to obtain the body mass index (BMI) which was calculated as the weight in kilograms divided by the squared value of the height in meters.

Ethical clearance was obtained from the Akwa Ibom State Ministry of Health Ethics and Research Committee (Number MH/PRS/99/V.IV/33). Permission was obtained from the Akwa Ibom State Ministry of Health to conduct the study at the health centers. Informed verbal consent was obtained from study participants and all data were kept confidential and anonymous.

### 2.3. Survey Instrument

A purposive questionnaire containing the following modules was used to collect the data for the study: demographics characteristics, smoking habit, perspective exposure measures, subjective health status (functional capacity limitation), disease symptoms inventory, risk perception, worry status, environmental risk tolerance, environmental annoyance and health symptoms inventory. The scales are described in greater detail below as they include novel features that are specific to chronic environmental adversity associated with oil pollution. Most of the descriptive items (descriptors) in this study were scored on a four-point scale instead of the usual 5-point or 7-point system used in other studies [18]. A significant number of the participants had low educational attainment and would be challenged to make the fine distinction on a scale with too many gradations. A large number of participants chose to do the survey in their local languages which made it even more semantically difficult to have more scale values. The scales used in this study make it possible to explore, for the first time, the multidimensional aspects of exposure and how these influence the health outcomes.

*Functional Capacity Limitation (FCL)*. For this study, we used one of the constructs in the general health questionnaire (GHQ) designed to measure limitations in one’s activities during a typical day [19,20,21]. Nine of the 11-item version of the GHQ for measures of functional capacity limitation were used in this study and seven of these were modified to address typical activities for the study population; we included going to the market, fishing and farming, for instance. Participants were asked to rate the extent to which a particular activity was limited during a typical day. The following (remaining) four questions from the classic GHQ were used basically unchanged: “*How much time during the past four weeks have you felt calm and peaceful?*”, “*How much of the time during the past four weeks did you have a lot of energy?*”, “*How much time during the past four weeks have you felt down?*”, and “*During the past four weeks, how much of the time has your physical health or emotional problems interfered with your social activities like visiting with friends, relatives, etc.?*”. Each item was scored on a four-point scale: (1) less than usual; (2) no more than usual; (3) rather more than usual; or (4) much more than usual. The sum of the scores of the 8 items was used as a subjective measure of health status for each participant. Total score for the scale (range, 4 to 36) was classified into 4–9 (low), 10–20 (medium) and 21–36 (high).

*Environmental Exposure Questions (EEQs)*. We used four environmental exposure questions to obtain perspective measures of each participant’s exposure to oil pollution in terms of residential distance from sources of exposure and frequency of direct contact with oil pollution. The first questions (EEQ1) asked how far a participant’s house was from visible oil pollution with the answers to be selected from being <50 m, 50–100 m, 100–500 m and >500 m. The second question (EEQ2) asked for the distance of participant’s dwelling from gas flaring facilities with the choice of answers the same as in the first question. The third question (EEQ3) consisted of “In the past one month, how many times have you been exposed (come into direct contact with) oil pollution? The scaled answers were 1 (“never”), 2 (1–5 times), 3 (“5–10 times”), and 4 (“over 10 times”). In the final measure (EEQ4), each participant was asked to rate the level of oil pollution in his/her drinking water on a scale of 1 (not contaminated) to 4 (highly contaminated). These exposure measures covered different dimensions of oil exposure and hence were treated as independent explanatory factors in the analysis—instead of summing the scores for the four questions to get a unitary measure of exposure for each participant. 

*Perceived Environmental Risk (PER)*. A variety of psychometric scaling methods have been developed to produce quantifiable and predictable measures of perceived environmental risk and perceptions for different types of hazards [22,23,24]. The scale used in this study was based partially on the risky events self-report questionnaire of Lerner *et al*. [25]. Each participant was asked to rate the likelihood that s/he would experience each of seven risky events that could be associated with oil pollution. The scale was introduced by the following question: “*On a scale of 1 (least likely or no concerns) to 4 (most likely), how strongly do you believe that the following will happen to you*?” Three of the items concerned a fire (getting hurt if the oil pollution catches fire; someone else sets the oil on fire; home and property damaged by such fire). Other items included getting sick from oil pollution; being a victim of violent crime because of issues around oil production; farm might be ruined by oil pollution; and the livelihood might be ruined by oil pollution. The range of total score for the scale (4–28) was categorized into 4–10 (low), 11–19 (medium), 20–28 (high).

*Environmental Risk Tolerance (ERT)*. This scale was patterned after the construct used in a recent study by Weiner *et al.* [18]. The participant was asked to rank each of 11 statements pertaining to local oil pollution on a scale of 1 (not bothered or concerned) to 4 (greatly bothered or concerned). The list of potential items that participants could find aversive in their areas and be reasons for concern included oil-polluted water, soil and fish contamination with oil, destruction of farmland, construction noise, germs caused by oil pollution, flaring of oil fields, destruction of fishing areas, increased floods and tides, number of strangers in the community and local air pollution. Total score for the scale was obtained by summing the numbers associated with the participants’ answers across all 11 items for a range of possible scores of 4–44. The scale was sub-categorized into 0–5 (minimal tolerance), 6–15 (low), 16–25 (medium) and 26–44 (high tolerance). The reverse score on the scales gives an indication of environmental risk intolerance.

*Environmental Hazard Annoyance (EHA).* Annoyance is a multidimensional concept that has variously been defined as a perception, an emotion, an attitude or a mixture of these [26]. The scale for this study was based on a set of semantic descriptors used in previous questionnaires to assess the degree of annoyance from sick-building syndrome, community noise, outdoor odors, industrial effluents, smoke from wood burning and similar stressors [26,27,28,29]. The questions were then modified appropriately to make oil pollution as the source of annoyance. The instrument for EAS used in this study is closely related to the constructs by Robin *et al*. [28] and Weiner *et al*. [18]. The module consists of 12 general examples of specific adverse environmental events related to oil pollution that probe the respondent’s level of annoyance and includes both positive direction and negative direction items. All items were scored on a scale of 1–4 scale (1 = this is not applicable to me; 2 = this disturbs me a little; 3 = I am moderately disturbed by it; and 4 = this disturbs me a lot). The negatively worded items were reverse scored and added to the remaining items to obtain a total score that ranged from 4 to 48. Total score for the scale was categorized into 4–10 (minimal), 11–20 (low), 21–30 (medium), and 31–48 (high) for the data analysis.

*Environmental Worry (EW).* The Penn State Worry (PSW) questionnaire [30] is currently the most frequently used instrument to assess pathological worry in both clinical and non-clinical populations [31,32]. This 16-item inventory assesses the intensity and excessiveness of worry without regard to its specific content. We adapted the PSW instrument by modifying six items to focus them specifically on tendency to worry about oil pollution in the study area. We removed two of the PSW items that did not seem relevant to the culture of the community being studied. The 14-item scale used in this study which included positive direction and negative direction statements was scored on a scale of 0 (no worry) to 3 (worry a lot). The potential total score of 52 was sub-divided into 4–10 (minimal), 11–20 (low), 21–35 (medium), and 36–52 (high).

*Health Symptoms Inventory (HSI)*. This module was designed to assess the burden of disease symptoms in the participants’ households. It is based on answers to the following question: “*Please tell me if you or anyone who lives in your household has experienced any of the following symptoms in the past six months?*” We used the household (family) data to gain a handle on the predictor symptom burden from chronic environmental contamination in comparison to personal factors associated with psychological distress (worry and annoyance) at the individual level. The 44-item scale for this study consisted of symptoms that are typically associated with environmentally-related diseases that generally account for more than 90% of the physical complaints reported in outpatient settings [33]. The symptoms pertain to the diseases of the eye (three items), nose and ear (four items), mouth (five items), throat (two items), respiratory system (three items), nervous system (14 items), stomach and bowel (six items). Responses were summed for each individual resulting in total score that ranged 0 to 44. The score was sub-divided into 0–5 (minimal), 6–15 (low), 16–25 (medium), and 26–44 (high).

### 2.4. Sample Size and Data Analysis

The sample size for this study was computed using SurveyMonkey [34]. The assumptions were that the total population in five LGAs used in the study was >350,000, the confidence level was 95% and margin of error was 5%. This sample size calculator which uses a normal distribution (50%) method yielded an optimum sample size of 384. To allow for a non-response rate of 20%, the final sample size determined was 461. In order to ensure a stable statistical model during analysis, the number of individuals recruited for the study was 600, with equal number drawn from each of the five LGAs.

All questionnaires were checked for completeness and coded prior to data entry on a laptop using an Excel spreadsheet. Data validation was done by taking a random sample of 10% and cross checking the entries on the spreadsheet with the corresponding questionnaires. The final spreadsheet contained observations for 600 participants and was imported into SPSS version 21 (IBM Corp.: Armonk, NY, USA) for analysis. Preliminary descriptive analysis was done to identify any outliers, determine the normality in distribution of continuous variables, and identify any patterns in the data. Bivariate analysis was done using Pearson’s chi square test to examine the correlations between demographic variables, exposure measures, and scores for risk perception, emotional distress, and health outcomes. Multiple regression analysis was used to explore the relationships between the outcome measures of interest and the influencing factors controlling for potential confounding variables. Finally, four-step hierarchical logistical regression models were used to analyze differences between explanatory factors grouped into: (i) emotional distress; (ii) perception of risk; (iii) measures of exposure; and (iv) demographic characteristics. In each model, associations of the explanatory factors with health outcome measures were estimated while adjusting for the covariates. Models were assessed for multicollinearity through examining the variance inflation factor (VIF), and no multicollinearity was found. In multivariate analysis of variance (ANOVA), Tukey’s *post-hoc* test was used for multiple comparisons of the within group effects. Two-tailed *p*-values are presented throughout and significance was assumed at the 95% confidence limits of the effect estimates.

## 3. Results

Table 1 summarizes the demographic characteristics and smoking habits of the study population. A great majority of participants (88%) fell into the 20–49 age range, reflecting the fact that the recruiting strategy used in the study targeted parents and healthy adults at the clinics. The dominant ethnic groups in the study were the Ibibios (46%) and Orons (30%). Most of the participants were either married (60.2%) or single (27%) and the proportions that were divorced (4%) or widowed (6.5%) were surprisingly small. Educational attainment was evenly split between secondary education (26%), post-secondary education (26.8) and professional education (27.5%), and only 7% of the respondents had primary school education. Only 28% of the respondents had formal employment in spite of the high level of education—high unemployment was one of the underlying factors in civil unrest in the communities studied. The proportion of the respondents that smoked regularly was 17% (Table 1).

Reference data on body mass index (BMI) for the Nigerian population does not currently exist. Viewed in relation to the BMI data for the U.S. population, it would appear that 4.3% of the respondents were severely underweight, 6.5% were underweight, 51.5% had normal weight, 25.5% were overweight and 12.3% were obese (Table 1). The fact that only about 11% were underweight and 38% were overweight suggests that any effects of oil pollution on local food supplies do not translate into undernutrition in the local communities.

Most of the respondents (86%) live <500 m from visible oil pollution with nearly half (48%) living within 100 m from some type of visible oil pollution (Table 1). A large majority of the respondents (73%) also lived <500 from a gas flaring facility with 48% living less than 100 m from gas flares (Table 1). Most participants came into direct contact with oil pollution fairly frequently with 53% reporting 1–5 contacts per month and 40% reporting 5–10 contacts per month. Contact with oil could come during bathing, washing clothes or fishing in oil-contaminated waters, farm work on oil-contaminated soils, and from job-related exposures. A large majority of participants (77%) perceived the drinking water to be highly contaminated while only 1% believed that drinking water was not contaminated with any oil (Table 1). These results show that most of the participants lived in areas with visible oil pollution and/or near gas flaring facilities and regularly suffered direct exposure to oil in their environment.

Emotional distress associated with oil pollution was very prevalent among the study participants. Only <5% of the respondents had no adverse feelings about oil pollution in the community (Table 2). By contrast, 81% were very worried, 86% were very angry, and 72% were very fearful and a large majority (68%) was very frightened or very stressed (66%) about local oil pollution (Table 1). These data show that emotional distress in varying degrees is a part of everyone's life in the area studied.

The Cronbach’s alpha value (a common matric for estimating the reliability of psychometric tests) for instrument is shown in Table 2. With the exception of the environmental hazard annoyance (EHA), the alpha values of the scales for the exploratory factors are over 0.80 implying a high degree of reliability. The descriptive statistics for the various measures of health outcomes (functional capacity limitation or FCL and health symptoms inventory or HSI) along with the scores for PER, ERT, EW, and EHA scales are also shown in Table 2 along with the expected range of scores for each scale. The mean (16.1 ± 5.1) and median (14.0) values for FCL are low compared to the maximum possible score (48), implying that the daily activities of the participants were not being severely limited by exposure to local oil pollution. However, the mean score for health symptom inventory (HSI) was 32.7±11—a high symptom burden considering that the maximum possible total was 43. The data for FCL and HSI are consistent with the result for the general health question where 36% of the participants reported their health to be “good” or “very good” and 58% reported their health to be “fair”. Only 5.5% reported their health to be “poor”. The mean values for EW (37.3±3.5), ERT (14.7±4.2), PER (26.5±2.7), and EHA (38.6±3.4). The ratio of the mean value to maximum possible value gives the following indication of the prevalence rates for the explanatory factors: 67% for EW, 33% for ERT (conversely, 67% for environmental risk intolerance), 80% for EHA and 95% for PER. These data show high prevalence rates for these psychopathological traits in the study population and a great awareness of oil pollution as a health hazard in the community.

The pattern of inter-relationships between the exposure measures was consistent with what one would have expected (Table 3). The strongest correlations were found between residential distances from gas flaring sites (EEQ1) and visible oil pollution (EEQ2) (*r* = 0.515, *p* < 0.001), and between EEQ3 and EEQ4 pair (*r* = 0.281, *p* < 0.001). Other exposure pairs that were significantly correlated were between EEQ1 and EEQ4 (*r* = −0.225, *p* < 0.0021), and EEQ2 and EEQ4 (*r* = −0.127, *p* < 0.001). These inter-relationships show that the measures reflect different dimensions of exposure to oil pollution in the study area.

The exposure metrics were significant predictors of the health effects and influencing factors (Table 3). The strongest correlations were found for the scores of EEQ2/HSI pair (*r* = 0.600, *p* < 0.001); EEQ2/EW (*r* = 0.46; *p* < 0.001); EEQ2/PER (*r* = 0.41, *p*-value < 0.001); and inversely with EEQ2/ERTS pair(*r* = −0.48, *p*-value < 0.001). The score for EEQ2 was not associated with environmental annoyance (*r* = 0.03, *p*-value = 0.74). Oil contamination of water (EEQ4) was significantly correlated with all the exploratory variables (Table 4) with the strongest correlations being with PER (*r* = 0.40, *p* < 0.001); HSI (*r* = 0.27, *p* < 0.001); EW (*r* = 0.27, *p* < 0.001); and inversely with ERT (*r* = −0.41, *p* < 0.001).

These data suggest that most of the respondents regarded gas flaring facilities and contamination of drinking water as the main health hazards related to oil pollution in the community. Living near areas with visible oil pollution (EEQ1) was significantly correlated with the explanatory factors, the only exception being environmental risk tolerance (*r* = 0.01, *p*-value = 0.74). None of the correlation coefficients for EEQ1 exceeded 0.3 however. The correlations with EEQ3 were even weaker with none of the *r*-values for the influencing factors and health effects exceeding 0.2 (Table 4). These data show that the participants mostly associated the negative health effects from oil pollution to be in the order: (gas flaring facility) > (oil contamination of drinking water) > (visible oil pollution) > (direct oil contact). The fact that the measures of oil exposure used in this study are positively correlated with perceptions of environmental contamination as well as health effects show that the measurement scales provide a meaningful tool in the current assessment. The results emphasize the importance of using multiple exposure measures in assessing the multi-dimensional health effects of oil pollution.

Table 3 shows the correlations of the explanatory factors themselves with health effects. All the influencing factors were significantly associated with each other with the strongest correlations found for PER/ERT (*r* = −0.775, *p* < 0.001), PER/EW (*r* = 0.557, *p* < 0.001), and EW/ERT (*r* = −0.467, *p* < 0.001). Likewise, all the influencing factors were significantly associated with FCL and HSI with the strongest correlations being HSI/PER (*r* = 0.561, *p* < 0.001), HSI/ERT (*r* = 0.544, *p* < 0.001), and HSI/EW (*r* = 0.528, *p* < 0.001). 

The strongest association of any of the explanatory factors with FCL was with PER (*r* = 0.334, *p* < 0.001). The correlation between HSI and FCL was modest (*r*^2^ = 0.119), indicating that the two scales provide independent measures in the exposure-health outcome relationships. None of the correlations in these analyses rose to the level of autocorrelation (*r* > 0.8), hence the explanatory factors were used as independent predictors in subsequent models. Multivariate analysis of the association of perspective measures of exposure with the health outcome and with the explanatory factors are shown in Table 4 and Table 5 respectively. All the exposure pathways were significantly associated with the HSI with the strongest association being with residential distance from gas flares (*r*^2^ = 0.36). All but one exposure source (residential distance from visible site of oil pollution) was not associated with FCL (Table 4). Direct contact with oil pollution (EEQ3) was not associated with any of the explanatory factors (Table 5). In addition, EEQ1 was not significantly associated with the score for environmental risk tolerance. The strongest associations of the explanatory factors were with residential distance from gas flares (Table 5).

The effects of demographic variables, explanatory factors and smoking habit on the health outcomes are shown in Table 6. These latent variables were able to explain 52% of the variance in HSI and 31% of the variance in FCL. The demographic variables were mostly associated with FCL whereas each explanatory factor was significantly associated with HSI (Table 6). BMI was not associated with any of the health measures but smoking cigarette was associated only with the score for health symptoms inventory. The interaction of explanatory factors had a significant mediating influence mainly on HSI (Table 6). A multi-level analysis of the effects of emotional distress (EW, EHA and ERT), perception of risk, demographic factor and smoking habit on FCL and HSI scores are shown in Table 7. From the model summaries, these factors together were able to explain 65% of the variance in somatic symptoms and 54% of the variance in functional capacity limitation. Model prediction improved marginally from Level 1 consisting of the WS, EAS and ERT scores (*r* = 0.63) to Level 4 consisting of all the explanatory factors (*r* = 0.65) for HSI, and from 0.47 to 0.54 for functional capacity limitation. Emotional reactions (EW, ERT and PER) together accounted for 39% of the variance in HSI, risk perception for 1.5% while demographic variables and smoking habit explained less than 1% of the variance in HSI. Model summary for FCL showed that emotional reactions (22%) and demographic variables (7.1%) accounted for most of the variance whereas the influence of risk perception and smoking are minimal (<1% combined).

## 4. Discussion

Nigeria flares the second largest amount of natural gas in the world (after Russia) and accounts for 10% of the total amount flared globally [35]. Flaring has been reduced to about 21% (or about 18 billion cubic meters in 2013) of the gas production, although most (86%) of the gas produced by marginal field operators and sole risks/independent operators is still flared [36]. Generally, gases from chemical factories, oil refineries, oil wells, rigs and landfills, gaseous waste products and sometimes even non-waste gases produced are routed to an elevated vertical chimney and burnt off (flared) [37]. Gas flaring facilities are often located close to local communities and typically lack adequate fencing or protection for villagers who regularly get exposed to the heat of the flare in their daily activities. Flares in many areas are continuous for 24 h a day thus subjecting the residents to continuous noise pollution and permanent light. When gas is flared, the combustion is often incomplete leading to formation of large amounts of soot and black carbon (black smoke highly enriched in polycyclic aromatic hydrocarbons) that are deposited on nearby land, buildings and properties and inhaled by local residents. Gaseous pollutants from gas flaring include nitrogen oxides, sulfur oxides, carbon monoxide and carbon dioxide, hydrocarbons and photochemical oxidants which can have pungent odor. The combination of noise, odor, heat, continuous night lighting, and black smoke no doubt have instilled a heightened sense that these emissions are not good for one’s health [38,39]. Above all else, constantly seeing the burning gas (fire) makes it more of a threat and thus more to be feared and not easily ignored. Luginaah *et al*. [40] made the important observation that catastrophic fears (such as explosions and fire), together with visual cues (including flares and smoke stacks) create considerable emotional distress, compounded by the uncertainty about the possible health impacts of what is in the “smoke”. Their observation is clearly evident with gas flares in the Niger Delta.

Studies in many parts of the world suggest that living near oil spills and petroleum production sites is an environmental stressor that can have adverse effects on health, well-being, and quality of life [9,41,42,43,44,45,46,47]. In general, oil spills/pollution can influence human health through two complementary pathways: (a) exposures to the inherently hazardous chemicals such as para-phenols and volatile benzene from the oil which can directly impair health through systemic toxicity; and (b) indirect pathways that work through the perceptions of risk, worry, annoyance, and chronic stress that moderate the sequelae of poor health outcomes. Studies of oil spills in many parts of the world have reported on major physiological health consequences of exposure to oil pollution which include abnormalities in hematologic, hepatic, respiratory, renal, and neurologic functions and the exposed individual may experience frequent asthmatic attacks, headache, diarrhea, dizziness, abdominal pain, back pain, and other symptoms [48,49,50,51,52]. These physiological effects almost always co-occur with emotional distress even where individuals are not directly exposed to the oil [5,53].

This study found high prevalence rates for symptoms that have been associated with oil spills in other parts of the world [6,48,49,50,51,52], including headache (96%), watery eyes (81%), sore throat (80%), respiratory problems (64%–83%), itchy skin (84%), rashes on face and neck (78%), sneezing, coughing or congested nose without a cold (83%), nausea (70%), dizziness (79%), chest pain (80%) and diarrhea (74%). Previous studies in oil polluted communities in the Nigerian delta have likewise reported high prevalence rates for various symptoms including headache, nausea, diarrhea, sore eyes, sore throat, cough, itchy skin, rashes, respiratory problems, and general malaise (see [6] for instance). The prevalence rates found in this study are comparable to or higher than reported in other studies in the country because our instrument was designed to measure the symptom burden in each participant’s household during the six months prior to the study; we show that the disease burden in a participant’s household is higher than that for the particular individual. As in many other previous studies [5,6,9,10,11,12], we found the symptoms to be correlated with the measures of exposure. The HSI score was found in this study to be significantly correlated with the distance of participant’s residence from visible oil or EEQ1 (*r* = −0.213, *p* < 0.001); distance of participant’s residence from gas flaring facility or EEQ2 (*r* = −0.60, *p* < 0.001); self-reported direct contact with oil pollution or EEQ3 (*r* = 0.185, *p* < 0.001); and oil pollution in drinking water or EQ4 (*r* = 0.271, *p* < 0.001). The FCL was also significantly correlated with all the exposure measures (Table 3). These results suggest that the health effects are indeed linked to perspective measures of exposure (EEQ1 and EEQ2) and measures of direct contact with oil (EEQ3 and EEQ4). These results also suggest that exposure sources are contributing directly to the burden of HSI and FCL in the communities that were studied.

### 4.1. Pathological Symptoms versus Functional Capacity Limitations

Oil production and refining consume and produce potentially harmful substances some of which are toxic, foul smelling, or flammable and hence give rise to worry, annoyance and concerns about the health consequences of exposure to such environmental contaminants [54]. Currently, the functional significance of exposure to such toxic components of crude oil in the etiology and maintenance of somatic symptoms are unknown. Some studies have suggested that chemical hypersensitivity and sensory irritation are a common phenomenon upon exposure to common odorants (including oil-related contaminants) in the environment [46,55]. In these studies, the health symptoms were found to be inducible by exposure to volatile organic compounds at levels below the threshold that can cause toxicological effects or sensory irritation in the eye, nose or throat [56,57,58]. For this report, we used multivariate models to explore possible latent effects of such noxious emissions on the inventory of health symptoms (HSI) as dependent variable and the four subjective measures of exposure as independent variables (Table 3).

The MANOVA result using Wilk’s lambda test was [P(4, 595) = 30.4, *p* < 0.001] and the multivariate *r*^2^ = 0.433 shows that the four exposure variables overall are a good predictors of HSI. The fact that exposure measures can account for 43.3% of the variance in HSI suggests that they are strong influencing factors on the health outcomes. The univariate test results (ANOVAs) for each exposure variable (Table 4) show that the dominant latent variables were residential distance from gas flare facilities (β = −7.7, *t* = −18.3, *p* < 0.001, *r*^2^ = −0.360) and drinking water with oil pollution (β = 3.6, *t* = 5.8, *p* < 0.001, *r*^2^ = 0.054). Both residential distance from visible oil pollution (*r*^2^ = 0.035, *p* < 0.001) and direct contact with oil (*r*^2^ = 0.019, *p* = 0.001) were also significantly associated the HSI score. These results are consistent with the claim in a number of studies that the proximity of environmental health hazards in itself can act as a stressor that affects the psychological and physiological responses [40,59,60,61].

Subjective health status (self-rated health) measured with an appropriate construct has repeatedly been shown to be a valid estimate of an individual’s biological, psychological and social dimensions of health [62,63,64,65]. The SHS has been shown to be a good predictor of morbidity and mortality in a wide range of populations [66,67,68] and has been related to important medical pathologies including diabetes mellitus, cardiovascular disease and history of cancer and depression [21,69] in adult populations. The SHS scale used in this study was focused specifically on the effect of exposure to oil pollution on the functional capacity limitation (FCL) of individuals in local communities. The FCL was moderately correlated with the HSI score (*r* = 0.34, *p* < 0.001), implying that the two scales are indeed measuring different dimensions of the health status. The relationships between concerns on the effects of oil pollution in making one sick *versus* making one’s family sick would be an interesting area for further research.

We next used regression models to explore the relationships between FCL and the four exposure metrics. Compared to the symptoms inventory, the four exposure factors were less able to predict the FCL with the r^2^ for the MANOVA result being 0.102 (compared to 0.433 for HSI). The univariate test results for individual exposure variables show that oil pollution in drinking water (EEQ4) was the strongest predictor but could only account for about 5% of the variance in FCL. The EEQ2 was the next significant influencing factor on FCL (*r*^2^ = 0.03, *p* < 0.001) and EEQ1 was not significantly associated with activity limitation. The four perspective measures of exposure could account for just about 10% of the variance in limitation of participant’s activities due to ill health, well below the main effect of exposure sources on HSI (43%). These results lead to suggestion that participants were mostly concerned about the impacts of exposure sources on their family (children was likely) and that the exposure sources have less influence on pathologies that limit their daily activities.

### 4.2. Perception of Oil Pollution Risks

Risk perception, the way people approach, think about, and interpret the risks in their environment is a multidimensional construct which is often associated with psychological distress and a range of other effects on health and well-being [70,71,72,73]. Although research on risk perception has a history of nearly half a century in North America, Europe and Asia [74,75], there has been no in-depth study of how the people of the Niger Delta appraise the environmental risks. It is probably fair to assume that most of the people in the Niger Delta do not have sufficient knowledge of science and technology to be capable of judging risks (typically based on the demonstrable probability of coming to harm, together with the severity of possible outcomes), costs and benefits quantitatively [76]. At the community level, an appropriate presentation of risk perception is likely to be along the line of the so-called *psychometric paradigm* [22] which represents environmental hazards in two dimensions defined by Unknown Risk factors and Dreaded Risk factors [75,77]. We hypothesize that the risk perception in the study area is mediated, to a large extent, by dreaded hazards (catastrophic fears) such as pipeline explosions and oil spill fire), visual cues (especially gas flares and smoke stacks) and chemosensory cues (such as drinking water with off flavor).

To confirm this hypothesis, we ran regression models using the perceived risk scale (PER) index as response variable and the four sources of exposure risks as explanatory factors. We found that the perspective measures of exposure are a good predictor of the PER score (*r*^2^ = 0.295, *p* < 0.001). The ANOVAs for each exposure variable show that the dominant latent variables were residential distance from gas flare facilities (β(standardized) = −1.15, *t* = −10, *p* < 0.001, *r*^2^ = 0.144) and drinking water with oil pollution (β(standardized) = 1.64, *t* = 9.6, *p* < 0.001, *r*^2^ = 0.133). Both residential distance from visible oil pollution (*r*^2^ = 0.006, *p* = 0.068) and direct contact with oil (*r*^2^ = 0.002, *p* = 2.79) were not significantly associated with perceived risk in the community. The relationship of direct contact with oil and increased likelihood of direct contact with oil (residential distance for visible oil pollution) with PER is consistent with the important role that visual and chemosensory cues play in participants’ perception of risks of local oil pollution.

We posit further that risk perception in the Niger Delta communities is beholden to the age-old conviction that how we think about an event determines how we feel about it. This proposition is aligned with the appraisal theories of emotion [78,79] which postulate that the appraisals (or evaluations) an individual makes in a given situation determine both the type and intensity of emotion that a person will have in that particular situation. Among the most intense emotional reactions to oil pollution in the Niger Delta are annoyance (80% prevalence rate) and worry (67% prevalence rate). 

### 4.3. Environmental Hazard Annoyance (EHA) and Environmental Worry (EW)

Emissions from oil polluted areas and petrochemical plants can infuse the surrounding areas with odors and impart off-flavor to local water supplies [80]. In addition, oil production and refining and their associated means of transport (heavy vehicles, tank trucks, ships and helicopters) create chronic noise while the gas flares can lead to exposure to heat, perpetual light and air pollution. These stressors have been associated with perceived negative health conditions in residential areas located close to oil refineries or petrochemical industries in many parts of the world [40,41,42,43,59,81,82,83]. Multivariate models with EW, PER, EHA and ERT as dependent variables and perspective measures of exposure as covariates (Table 5) show that EW is more strongly associated with exposure variables (*r*^2^ = 0.27; *p* < 0.001) compared to EHS (*r*^2^ = 0.08, *p* < 0.001). ANOVA for individual exposure variables show that residential distance from gas flares was the dominant cause for worry whereas the actual contacts with oil pollution (EEQ3 and EEQ4) were more strongly associated with hazard annoyance (Table 5). Our data leaves no doubt that oil pollution is an environmental stressor that induces a lot of worry and annoyance in local communities.

Annoyance can be defined as a feeling of displeasure associated with any agent or condition believed to have an adverse effect [83] and is often attended by other negative emotions such as feelings of irritation, frustration, dissatisfaction, discomfort, distress, anger, fear, and hatred [84]. It is a key antecedent and a strong predictor of *rumination* which may be broadly described as a tendency to continue to think about something bad, harmful, or unhopeful for a long time [85]. Annoyance from environmental exposure has been suggested to serve as an early warning signal of illness [86,87]. Worrying can be defined as a relatively uncontrollable chain of negative thoughts and images [88]. Worry and rumination (and its antecedent annoyance) are not the same, however, but overlapping forms of what is called perseverative cognition [89,90]. In terms of environmental exposures, the content of rumination may be condensed into the thought process (repetitive, intrusive) as well as thought content (negative cognitions) about the environmental adversity whereas the content of worry tends to condense into the theme of anticipated threat (risk perception) about the environmental hazards [91]. Data from this study (Table 3) yielded a moderate association between the scores for the measures of environmental annoyance and environmental worry (*r* = 0.36, *p* < 0.001), indicating that these two forms of emotional reaction represent different components of the emotional distress continuum in the communities studied.

### 4.4. Tolerance to Oil Pollution Risk

How a person tolerates an environmental hazard will impact how often s/he worries or ruminates about it and therein lies their level of chronic stress [92,93,94]. In general, people who are intolerant of environmental hazards are likely to interpret all ambiguous risk information as threatening [95], contributing to significant emotional reactions. In contrast to such risk aversion, risk tolerance is a developed characteristic that allows one to sleep at night without stressing over oil pollution and related environmental issues. We consider environmental risk tolerance (ERT) to be an important coping strategy which has not been explored previously in communities of the Niger Delta.

The mean ERT score for the study population (14.7 ± 4.2 out of total possible score of 44) was low, implying a high level of intolerance to oil pollution in the community. The ERT score was not significantly correlated with age, ethnic origin, or marital status of the participants. Age-based risk tolerance is a cliché based on the fact that younger people have long-term expectation that things will improve and are hence more risk tolerant whereas older people generally have lower risk tolerance. This wisdom does not apply to our study site where people are exposed to and made aware of the oil pollution around them at an early age. The ERT score was inversely correlated with educational level (*r* = −0.101, *p* = 0.013), implying that knowledge increases the level of intolerance to oil pollution in the community.

We found that score for perceived risk was strongly correlated with the score for environmental worry (*r* = 0.557, *p* < 0.001) but less strongly with the score for environmental annoyance (*r* = 0.189, *p* < 0.001), and both worry and annoyance scores are significantly inter-related (*r* = 0.356, *p* < 0.001) (Table 3). These results show that risk perception may be a predictor of both environmental worry and environmental annoyance and consequently may enhance the level of comorbidity between the emotional disorders of annoyance and worry in the study population [96]. All three explanatory factors are strongly correlated with the health symptoms inventory (Table 3). These results are consistent with reports by other authors who have found risk perception, environmental annoyance and environmental worry to be influencing factors on the burden of disease symptoms in areas of large oil spills [49,97,98,99].

Multivariate analysis with the four influencing factors as dependent variables and the four exposure sources as independent variables showed that perspective measures of exposure could account for 35% of the variance in ERT. The association was mainly due to the effects of two sources (Table 5): positively with residential distance from gas flares (β = 2.01, *t* = 11.8, *p* < 0.001; *r*^2^ = 0.19) and negatively with oil in drinking water (β = −2.65, *t* = 10.5, *p* < 0.001; *r*^2^ = 0.16). The observation that risk tolerance increases with residential distance from gas flaring may be related to the dramatic local effects within the plume of gas stacks (from high levels of dust fall and gaseous air pollutants to lighting of the night) which decreases with distance away from such effects. In terms of correlation with EEQ4 (*r* = −0.41, *p* < 0.001; Table 3), it should be noted that oil gives objectionable taste and odor to drinking water which are likely to elicit a strong emotional reaction to the contaminant and its source. The distance from visible source of oil pollution (*r*^2^ = 0.002, *p* = 0.313) and direct contact with oil (*r*^2^ = 0.002, *p* = 0.33) had no significant effect on ERT. The later results are consistent with the hypothesis that the presence of a potentially hazardous facility in close proximity to a residential community generates a constant risk signal that conditions and desensitizes that population thereby increasing their risk tolerance [18,100]. While people in a stressed community may be tolerant of particular environmental hazards (oil polluted soils and water in our case), they can become very intolerant when the hazards include strong negative visual or sensory cues, however.

### 4.5. Risk Perception and Emotional Distress as Modifiers of the Association between Exposure to Oil Pollution and Health Effects

Semantically, risk perception is sometimes measured by means of judgments about worry and in everyday language, worry and risk are often used interchangeably [91,101]. Our results show that worry is strongly associated with risk perception (*r* = 0.557; *p* < 0.001), but the two variables are not auto-correlated (*r* < 0.8). We used the data from this study to assess the associations between perception of risk, annoyance and worry and how these relations mediate the health symptoms index (HIS) and functional capacity limitation (FCL) within the community context (Table 6).

The relationships between the explanatory factors (ERT, EW, PER and EAS scores) as independent variables and health outcomes (HSI and FCL scores) were first explored using linear regression models. The effects of demographic confounders (age, ethnic origin, level of educational attainment, BMI) and smoking habits were included in the analyses (Table 6). Only the explanatory factors and smoking habits were significantly associated with HSI (Table 6).

The interactions of explanatory factors, namely EHA*ERT, ERT*PER, EW*ERT, and EW*PER were also significantly associated with HSI. None of these interaction factors was able to explain more than 3% of the variance in HSI (Table 6). By contrast, only the demographic factors (age, ethnic origin, educational level) and one interaction factor (EW*PER) were associated with FCL (Table 6). Environmental annoyance was not associated directly with the HSI (Table 5), but its interactions with EW and ERT suggests that its influence is indirect through mediation by risk intolerance.

We used step-wise regression models to explore the effects of key influencing factors on HSI and FCL. The factors were classified into two main dimensions: (i) emotional reactions and (ii) exposure sources and demographics. For these models, variables were progressively added to improve the estimated regression coefficient in each step. The multi-levels of analyses were as follows: Model I considered emotional reactions alone; Model II was Model 1 plus risk perception; Model III consisted of Model II plus exposure sources; while Model IV consisted of Model III plus demographic variables. These models show that the influencing factors on measures of activity limitation status (FCL) were different from those for somatic symptoms (HSI). For one thing, the main effect of the emotional reactions on FCL was much less (*r*^2^ = 0.219) compared to HSI (*r*^2^ = 0.394). Model I shows that participants who worried a lot reported more activity limitations (β = 0.56, *p* < 0.001; *r* = 0.334) and those with higher annoyance scores also had more activity limitations (β = 0.18, *p* = 0.002; *r* = 0.127). Participants’ environmental risk tolerance score did not significantly influence their FCL score. Although risk perception was significantly associated with FCL (β = 0.27, *p* = 0.022; *r* = 0.094) this factor only account for 0.7% of the variance in FCL (Model t; Table 7). Demographic variables (Model III) had much more significant influence on FCL; the change in *r*^2^ (Δ*r*^2^) with these factors in the model was 0.07 compared to HSI (Δ*r*^2^ = 0.002). Of some note is the fact that residential distance from gas flaring facilities was not even significantly associated with FCL (β = 0.01, *p* = 0.85; *r* = 0.008). Demographic factors (Model IV) could explain 7.3% of the variance in FCL compare to only 0.7% for HSI. Among the demographic factors, BMI was the characteristic that was not associated with HSI of FCL. Smoking habits had minimal and non-significant influence (β = 0.014, *r* = 0.016, *p* = 0.69) on FCL. These results suggest that at the individual level, the demographic variables and direct contact with oil pollution were the key determinants of the FCL. At the community level, the sources of exposure could lead to emotional distress that mediated the health symptoms.

## 5. Limitations

Although the results of our research are encouraging and useful, they are tempered by the limitations of the research. As the first limitation of our study, we must point out that the study relied solely on self-evaluation scales. The symptoms and functional capacity limitations were not ascertained or verified by independent means such as from medical chart or clinical examination. This may be true although the recent study by Peek *et al*. [60] found good agreement between the results of objective and subjective measures of exposure on psychopathological health effects. There was no pre-exposure data and very limited measure of objective exposure. Within the context of global environmental health research model, we used the best available recruitment methods to assemble the participant samples. Nevertheless, the sampling procedure (clinic based) is considered a limitation which might have led to sampling biases. The cross-sectional approach represents an additional limitation that precludes the ability to directly establish a causal relationship between exposure to oil pollution and any particular disease condition among the community members. We believe that the use of longitudinal designs would give stronger information on the processes underlying the associations found in the present study. Because of the multi-dimensional nature of the effectors, multiple comparisons have to be made in the analysis. It is possible that some of the statistically significant findings being reported may be due to chance given the multiple comparisons made (so-called Type II error in statistical practice). We believe that our sample size is large enough to minimize such errors in the models used in the analysis.

## 6. Conclusions

This study documents a high level of worry, annoyance and intolerance associated with oil production and refinery in the Niger Delta areas of Nigeria. Emotional distress can induce dysregulation of multiple interrelated physiological systems including the cardiovascular, endocrinological and immunological systems and hence are risk factors for a wide range of pathological diseases [60,89]; the high burden of disease symptoms found in this study is noteworthy. As example, people with type II diabetes mellitus, a rapidly rising condition in Nigeria, are twice as likely to suffer from depression compared to the general population, which in turn can lead to greater difficulty with self-care [102]. People with emotional distress are more likely to smoke cigarettes as other people [103,104]; we show that 17% of the participants smoked regularly and this rate may be rising. Patients who are depressed have higher the risk of having a heart attack compared to the general population [105]. Of particular significance in Nigeria is that fact that annoyance and intolerance are among the instantiations considered to be risk factors for aggression through loss of self-control [106]. People who show heightened aggressive cognition, physiological arousal, and anger tend to make hostile attributions about others’ behavior (polluters in the Niger Delta for instance), which heightens the tendency towards aggression [107]. The cause-effect relationship between exposure to oil pollution and high level of violence in the Niger Delta as well as the hostility towards the oil producing companies is an intriguing question for further research. The results of this study make a strong case for national attention to be paid to what our study has shown to be a silent epidemic of psychological problems in communities of the Niger Delta area.

## Figures and Tables

**Table 1:** Summary statistics for demographic and exposure characteristics at the study site.

Parameter	Category/Range	Percentage
Age	<20 years	2.7
	20–29 years	26.8
	30–39	38.9
	40–49	22.3
	50–59	7.3
	>60	1.8
Ethnicity	Ibibio	45.6
	Anang	4.2
	Oron	30.1
	Others	19.8
Marital status	Single/Never married	27.0
	Married	60.2
	Divorced	4.0
	Cohabiting	2.2
	Widowed	6.5
Educational Attainment	Primary School	7.0
	Secondary Sch	26.0
	Vocational/Techn	12.6
	Professional	27.5
	Post-secondary	26.8
Employment	Employed	27.5
	Un-employed	72.2
Use of cigarettes	Regular smoker	16.7
Body mass index (BMI)	Severely underwt (<16.5 kg/m^2^)	4.3
	Underwt (16.5–18.5)	6.5
	Normal (18.5–25)	51.5
	Overwt (25–30)	25.5
	Obese (>30)	12.3
Distance of residence from visible oil pollution	<50 m	7.2
	50–100 m	41.9
	100–500 m	36.3
	>500 m	14.1
Distance of residence from gas flaring facility	<50 m	6.2
	50–100 m	41.6
	100–500 m	25.1
	>500 m	26.8
Exposure to (contact with) oil pollution	Never	5.7
	1–5 times/mo	52.7
	5–10 times/mo	40.1
	>10 times/mo	1.3
Perception of oil contamination of drinking water	Not contaminated	1.0
	Slightly contaminated	3.3
	Contaminated	18.6
	Highly contaminated	76.9
Emotional distress about local oil pollution	Worried	81.0
	Fearful	72.0
	Frightened	68.0
	Angry	86.0
	Stressed	66.0

**Table 2:** Estimated values of the explanatory factor scores and the construct validity of each descriptor.

Statistic	Percentile	FCL *	EW *	ERT *	PER *	EHA *	HSI *
N		599	600	600	600	600	599
Mean		16.2	37.3	14.7	26.5	38.7	32.7
Median		14.0	37.0	14.0	28.0	40.0	37.0
Std. Deviation		5.2	3.5	4.1	2.7	3.4	10.9
Minimum		7.0	24.0	11.0	7.0	27.0	7.0
Maximum		28.0	49.0	44.0	28.0	47.0	43.0
Expected maximum value		44.0	56.0	44.0	28.0	48.0	43.0
Percentiles	25	14.0	35.0	12.0	26.0	37.0	22.0
	50	14.0	37.0	14.0	28.0	40.0	37.0
	75	20.0	40.0	16.0	28.0	41.8	43.0
Construct validity (Cronbach’s α)		0.82	0.81	0.82	0.87	0.51	0.95

* Acronyms: EW = environmental worry; PER = perceived environmental risk; EHA = environmental hazard annoyance; ERT = environmental risk tolerance; HSI = health symptoms inventory; FCL = functional capacity limitation; N = number of valid observations for a particular variable.

**Table 3:** Correlation of subjective measures of oil exposure with explanatory factors and health outcome measures.

Variable	Statistic	EEQ1 *	EEQ2 *	EEQ3 *	EEQ4 *	EW *	PER *	EHA *	ERT *	HSI *	FCL *
EEQ1	r	1	0.515 **	<0.001	−0.225 **	−0.164 **	−0.212 **	0.013	0.278 **	−0.213 **	−0.076
	*p*-value		<0.001	0.999	<0.001	<0.001	<0.001	0.743	<0.001	<0.001	0.062
	N	600	600	600	600	600	600	600	600	599	599
EEQ2	r		1	−0.033	−0.127 **	−0.456 **	−0.410 **	−0.027	0.480 **	−0.600 **	−0.188 **
	*p*-value			0.424	0.002	<0.001	<0.001	0.507	<0.001	<0.001	<0.001
	N			600	600	600	600	600	600	599	599
EEQ3	r			1	0.281 **	0.073	0.151 **	−0.060	−0.085 *	0.185 **	0.156 **
	*p*-value				<0.001	0.076	<0.001	0.141	0.037	<0.001	<0.001
	N			600	600	600	600	600	600	599	599
EEQ4	r				1	0.266 **	0.398 **	0.229 **	−0.409 **	0.271 **	0.257 **
	*p*-value					<0.001	<0.001	<0.001	<0.001	<0.001	<0.001
	N				600	600	600	600	600	599	599
EW	r					1	0.557 **	0.356 **	−0.467 **	0.528 **	0.450 **
	*p*-value						<0.001	<0.001	<0.001	<0.001	<0.001
	N					600	600	600	600	599	599
PER	r						1	0.189 **	−0.775 **	0.561 **	0.334 **
	*p*-value							<0.001	<0.001	<0.001	<0.001
	N						600	600	600	599	599
EHA	r							1	−0.133 **	0.128 **	0.264 **
	*p*-value								0.001	0.002	<0.001
	N							600	600	599	599
ERT	r								1	−0.544 **	−0.259 **
	*p*-value									<0.001	<0.001
	N								600	599	599
HSI	r									1	0.345 **
	*p*-value										<0.001
	N									599	598

** Correlation is significant at the 0.01 level (2-tailed); * Correlation is significant at the 0.05 level (2-tailed). *Acronyms*: EW = environmental worry; PER = perceived environmental risk; EHA = environmental hazard annoyance; ERT = environmental risk tolerance; HSI = health symptoms inventory; FCL = functional capacity limitation; EEQ1 to EEQ4 = scores for environmental exposure questions 1 to 4.

**Table 4:** Relationships between scores for subjective exposure to oil pollution and health outcomes.

Dependent Variable *		B	Std. Error	t	Sig.	95% Confidence Interval		Partial Eta Squared
						Lower Bound	Upper Bound	
FCL	Intercept	8.94	1.64	5.44	<0.001	5.72	12.17	0.048
	EEQ1	0.36	0.22	1.62	0.106	−0.08	0.79	0.004
	EEQ2	−1.09	0.25	−4.30	<0.001	−1.58	−0.59	0.030
	EEQ3	0.73	0.34	2.12	0.035	0.05	1.40	0.008
	EEQ4	2.03	0.38	5.39	<0.001	1.29	2.76	0.047
HSI	Intercept	30.99	2.75	11.26	<0.001	25.59	36.40	0.176
	EEQ1	1.71	0.37	4.62	<0.001	0.98	2.43	0.035
	EEQ2	−7.74	0.42	−18.28	<0.001	−8.57	−6.91	0.360
	EEQ3	1.97	0.58	3.42	0.001	0.84	3.11	0.019
	EEQ4	3.67	0.63	5.84	<0.001	2.44	4.91	0.054

* HSI = health symptoms inventory; FCL = functional capacity limitation; EEQ1 to EEQ4 = scores for environmental exposure questions 1 to 4; Std. Error = Standard error; Sig = Level of significance.

**Table 5:** Relationships between exposure sources of oil pollution and emotional reactions of the study population.

Dependent Variable *		B	Std. Error	t	Sig.	95% Confidence Interval		Partial Eta Squared
						Lower Bound	Upper Bound	
EW	Intercept	35.97	1.00	36.05	<0.001	34.01	37.93	0.686
	EEQ1	0.48	0.13	3.57	<0.001	0.21	0.74	0.021
	EEQ2	−1.88	0.15	−12.27	<0.001	−2.18	−1.58	0.202
	EEQ3	−0.06	0.21	−0.30	0.762	−0.47	0.35	<0.001
	EEQ4	1.45	0.23	6.36	<0.001	1.00	1.90	0.064
PER	Intercept	22.72	0.75	30.37	<0.001	21.25	24.18	0.608
	EEQ1	0.18	0.10	1.83	0.068	−0.01	0.38	0.006
	EEQ2	−1.15	0.11	−10.01	<0.001	−1.38	−0.92	0.144
	EEQ3	0.17	0.16	1.08	0.279	−0.14	0.48	0.002
	EEQ4	1.64	0.17	9.57	<0.001	1.30	1.97	0.133
EHA	Intercept	33.84	1.10	30.65	<0.001	31.67	36.01	0.612
	EEQ1	0.32	0.15	2.19	0.029	0.03	0.61	0.008
	EEQ2	−0.18	0.17	−1.05	0.293	−0.51	0.15	0.002
	EEQ3	−0.80	0.23	−3.45	0.001	−1.25	−0.34	0.020
	EEQ4	1.71	0.25	6.77	<0.001	1.21	2.21	0.072
ERT	Intercept	18.92	1.11	17.08	<0.001	16.75	21.10	0.329
	EEQ1	−0.15	0.15	−1.01	0.313	−0.44	0.14	0.002
	EEQ2	2.01	0.17	11.82	<0.001	1.68	2.35	0.190
	EEQ3	0.23	0.23	0.98	0.326	−0.23	0.68	0.002
	EEQ4	−2.65	0.25	−10.48	<0.001	−3.15	−2.16	0.156

* EW = environmental worry; PER = perceived environmental risk; EHA = environmental hazard annoyance; ERT = environmental risk tolerance; HSI = health symptoms inventory; FCL = functional capacity limitation; EEQ1 to EEQ4 = scores for environmental exposure questions 1 to 4; Std. Error = Standard error; Sig = Level of significance.

**Table 6:** Between-variable effects of demographic characteristics and explanatory factors on health outcomes.

Source		df	Mean Square	F	Sig.	Partial Eta Squared
Corrected Model *	HIS	15	2468.95	41.97	<0.001	0.520
	FCL	15	328.65	17.24	<0.001	0.308
Intercept	HIS	1	1063.50	18.08	<0.001	0.030
	FCL	1	29.07	1.52	0.217	0.003
EW	HIS	1	505.64	8.60	0.004	0.015
	FCL	1	59.79	3.14	0.077	0.005
EHA	HIS	1	585.51	9.95	0.002	0.017
	FCL	1	0.28	0.01	0.903	<0.001
ERT	HIS	1	755.96	12.85	<0.001	0.022
	FCL	1	13.08	0.69	0.408	0.001
PER	HIS	1	240.40	4.09	0.044	0.007
	FCL	1	14.70	0.77	0.380	0.001
BMI	HIS	1	44.37	0.75	0.385	0.001
	FCL	1	32.42	1.70	0.193	0.003
Age	HIS	1	21.19	0.36	0.549	0.001
	FCL	1	335.07	17.58	<0.001	0.029
Ethnicity	HIS	1	19.52	0.33	0.565	0.001
	FCL	1	246.05	12.91	<0.001	0.022
Education	HIS	1	0.16	0.00	0.959	<0.001
	FCL	1	113.54	5.96	0.015	0.010
EW*EHA	HIS	1	154.13	2.62	0.106	0.004
	FCL	1	9.33	0.49	0.484	0.001
EHA*ERT	HIS	1	555.84	9.45	0.002	0.016
	FCL	1	0.07	0.00	0.952	<0.001
ERT*PER	HIS	1	2107.48	35.83	<0.001	0.058
	FCL	1	19.44	1.02	0.313	0.002
EW*ERT	HIS	1	406.77	6.91	0.009	0.012
	FCL	1	13.14	0.69	0.407	0.001
EW*PER	HIS	1	282.54	4.80	0.029	0.008
	FCL	1	81.76	4.29	0.039	0.007
EHA*PER	HIS	1	191.34	3.25	0.072	0.006
	FCL	1	2.10	0.11	0.740	<0.001
Smoking	HIS	1	486.13	8.26	0.004	0.014
	FCL	1	4.04	0.21	0.645	<0.001

* The adjusted R Squared (partial eta squared) values for the corrected models are in the last column; *Acronyms*: EW = environmental worry; PER = perceived environmental risk; EHA = environmental hazard annoyance; ERT = environmental risk tolerance; HSI = health symptoms inventory; FCL = functional capacity limitation; EEQ1 to EEQ4 = scores for environmental exposure questions 1 to 4; BMI = body mass index; Sig = level of significance.

**Table 7:** Hierarchical analysis of the relationships between influencing factors and health outcomes.

Model	R	R Square	Adjusted R Square	Std. Error of the Estimate	Change Statistics				
					R Square Change	F Change	df1	df2	Sig. F Change
Health Symptoms Inventory (HSI) score									
1	0.628	0.394	0.391	8.51901	0.394	129.203	3	595	<0.001
2	0.640	0.409	0.405	8.42182	0.015	14.813	1	594	<0.001
3	0.647	0.419	0.411	8.38265	0.009	2.391	4	590	0.050
4	0.652	0.425	0.416	8.34617	0.006	6.168	1	589	0.013
Functional Capacity Limitations (FCL) score									
1	0.468	0.219	0.215	4.58700	0.219	55.504	3	595	<0.001
2	0.475	0.226	0.220	4.57054	0.007	5.294	1	594	0.022
3	0.545	0.297	0.287	4.37049	0.071	14.906	4	590	<0.001

Model 1: Constant, Environmental risk tolerance, Environmental Hazard annoyance, Environmental Worry; Model 2: Model 1 plus Perceived Environ Risk (PER); Model 3a: Model 1 + Model 2 + Age, Ethnic origin, Education, BMI; Model 4: Model 1 + Model 2 + Model 3 + Smoking habits; Std. Error = Standard error; Sig = Level of significance.
